# Supplementary material for: COVID-19 and the risk and trajectory of frailty in community- or institution-dwelling individuals: Protocol for systematic review and meta-analysis
Source: PLoS One. 2024 Nov 15;19(11):e0312163. doi: 10.1371/journal.pone.0312163 (PMC11567516; doi:10.1371/journal.pone.0312163)
Supplement: S1 Appendix — (DOCX) [file pone.0312163.s002.docx]

**S1 Appendix 1. Search strategy for all six databases**

**Topic:** Covid 19 and Frailty

**Covid-19 Filter Source:** <https://covid.cadth.ca/literature-searching-tools/cadth-covid-19-search-strings/>*

*Filter taken directly from CADTH for use in PubMed and Embase and combined with frailty terms**, but adapted to keyword searching (multi-colored by string) for Cochrane databases, SportDiscus, and CINAHL.

**The most simple terms were used for frailty based upon precedent set by a previous systematic review on the topic: <https://www.ncbi.nlm.nih.gov/pmc/articles/PMC5499023/>.

**PubMed**

((COVID-19 [mh] OR COVID-19 Testing[mh] OR COVID-19 Vaccines[mh] OR SARS-CoV-2[mh] OR spike glycoprotein, COVID-19 virus [Supplementary Concept] OR ((Coronavirus[mh:noexp] OR Betacoronavirus[mh:noexp] OR Coronavirus Infections[mh:noexp]) AND (Disease Outbreaks[mh:noexp] OR Epidemics[mh:noexp] OR Pandemics[mh])) OR nCoV[tiab] OR nCoV[tt] OR 2019nCoV[tiab] OR 2019nCoV[tt] OR 19nCoV[tiab] OR 19nCoV[tt] OR COVID19*[tiab] OR COVID19*[tt] OR COVID[tiab] OR COVID[tt] OR SARS-CoV-2[tiab] OR SARS-CoV-2[tt] OR SARSCOV-2[tiab] OR SARSCOV-2[tt] OR SARS-COV2[tiab] OR SARS-COV2[tt] OR SARSCOV2[tiab] OR SARSCOV2[tt] OR SARS coronavirus 2[tiab] OR SARS coronavirus 2[tt] OR Severe Acute Respiratory Syndrome Coronavirus 2[tiab] OR Severe Acute Respiratory Syndrome Coronavirus 2[tt] OR ((severe acute respiratory syndrome[tiab] OR severe acute respiratory syndrome[tt]) AND (corona virus 2[tiab] OR corona virus 2[tt])) OR new coronavirus[tiab] OR (new[tt] AND coronavirus[tt]) OR novel coronavirus[tiab] OR novel coronavirus[tt] OR novel corona virus[tiab] OR (novel[tt] AND corona virus[tt]) OR novel CoV[tiab] OR (novel[tt] AND CoV[tt]) OR novel HCoV[tiab] OR (novel[tt] AND HCoV[tt]) OR ((“19″[tiab] OR “19”[tt] OR “2019”[tiab] OR “2019”[tt] OR Wuhan[tiab] OR Wuhan[tt] OR Hubei[tiab] OR Hubei[tt]) AND (coronavirus*[tiab] OR coronavirus*[tt] OR corona virus*[tiab] OR corona virus*[tt] OR CoV[tiab] OR CoV[tt] OR HCoV[tiab] OR HCoV[tt])) OR longCOVID*[tiab] OR longCOVID*[tt] OR postCOVID*[tiab] OR postCOVID*[tt] OR postcoronavirus*[tiab] OR postcoronavirus*[tt] OR postSARS*[tiab] OR postSARS*[tt] OR ((coronavirus*[tiab] OR coronavirus*[tt] OR corona virus*[tiab] OR corona virus*[tt] OR betacoronavirus*[tiab] OR betacoronavirus*[tt]) AND (outbreak*[tiab] OR outbreak*[tt] OR epidemic*[tiab] OR epidemic*[tt] OR pandemic*[tiab] OR pandemic*[tt] OR crisis[tiab] OR crisis[tt])) OR ((Wuhan[tiab] OR Wuhan[tt] OR Hubei[tiab] OR Hubei[tt]) AND (pneumonia[tiab] OR pneumonia[tt]))) AND 2019/10/31:3000/12/31[Date – Publication]) AND ("Frailty"[Mesh] OR frail*[tiab])

**Embase**

1. sars-related coronavirus/
2. (coronavirinae/ or betacoronavirus/ or coronavirus infection/) and (epidemic/ or pandemic/)
3. (nCoV* or 2019nCoV or 19nCoV or COVID19* or COVID or SARS-COV-2 or SARSCOV-2 or SARS-COV2 or SARSCOV2 or SARS coronavirus 2 or Severe Acute Respiratory Syndrome Coronavirus 2 or Severe Acute Respiratory Syndrome Corona Virus 2).ti,ab,kw,hw,ot.
4. ((new or novel or “19” or “2019” or Wuhan or Hubei or China or Chinese) adj3 (coronavirus* or corona virus* or betacoronavirus* or CoV or HCoV)).ti,ab,kw,hw,ot.
5. (longCOVID* or postCOVID* or postcoronavirus* or postSARS*).ti,ab,kw,hw,ot.
6. ((coronavirus* or corona virus* or betacoronavirus*) adj3 (pandemic* or epidemic* or outbreak* or crisis)).ti,ab,kw,ot.
7. ((Wuhan or Hubei) adj5 pneumonia).ti,ab,kw,ot.
8. or/1-7
9. limit 8 to yr=”2019 -Current”
10. 'frailty'/de or frail*:ab,ti
11. 9 AND 10

**Cochrane CDSR**

**((**((COVID-19 OR SARS-CoV-2 OR Coronavirus OR Betacoronavirus) **AND** (Disease Outbreak* OR Epidemic* OR Pandemic*)) OR ((nCoV OR 2019nCoV OR 19nCoV OR COVID19* OR COVID OR SARS-CoV-2 OR SARSCOV-2 OR SARS-COV2 OR SARSCOV2 OR SARS coronavirus 2 OR Severe Acute Respiratory Syndrome Coronavirus 2 OR severe acute respiratory syndrome) **AND** ((corona virus 2 OR new coronavirus) OR ((new AND Coronavirus) OR (novel coronavirus OR (novel AND corona virus) OR novel CoV OR (novel AND CoV) OR novel HCoV OR novel AND HCoV))))) OR ((“19″ OR “2019” OR Wuhan OR Hubei) **AND** (coronavirus* OR corona virus* OR CoV OR HCoV OR longCOVID* OR postCOVID* OR postcoronavirus* OR postSARS* OR coronavirus* OR corona virus*OR betacoronavirus*) **AND** (outbreak* OR epidemic* OR pandemic* OR crisis)) OR ((Wuhan OR Hubei) **AND** (pneumonia)) **AND** (2019/10/31:3000/12/31[Date – Publication])**)** **AND** (frail*)**)**

**Cochrane CENTRAL**

**((**((COVID-19 OR SARS-CoV-2 OR Coronavirus OR Betacoronavirus) **AND** (Disease Outbreak* OR Epidemic* OR Pandemic*)) OR ((nCoV OR 2019nCoV OR 19nCoV OR COVID19* OR COVID OR SARS-CoV-2 OR SARSCOV-2 OR SARS-COV2 OR SARSCOV2 OR SARS coronavirus 2 OR Severe Acute Respiratory Syndrome Coronavirus 2 OR severe acute respiratory syndrome) **AND** ((corona virus 2 OR new coronavirus) OR ((new AND Coronavirus) OR (novel coronavirus OR (novel AND corona virus) OR novel CoV OR (novel AND CoV) OR novel HCoV OR novel AND HCoV))))) OR ((“19″ OR “2019” OR Wuhan OR Hubei) **AND** (coronavirus* OR corona virus* OR CoV OR HCoV OR longCOVID* OR postCOVID* OR postcoronavirus* OR postSARS* OR coronavirus* OR corona virus*OR betacoronavirus*) **AND** (outbreak* OR epidemic* OR pandemic* OR crisis)) OR ((Wuhan OR Hubei) **AND** (pneumonia)) **AND** (2019/10/31:3000/12/31[Date – Publication])**)** **AND** (frail*)**)**

**SportDiscus**

**((**((COVID-19 OR SARS-CoV-2 OR Coronavirus OR Betacoronavirus) **AND** (Disease Outbreak* OR Epidemic* OR Pandemic*)) OR ((nCoV OR 2019nCoV OR 19nCoV OR COVID19* OR COVID OR SARS-CoV-2 OR SARSCOV-2 OR SARS-COV2 OR SARSCOV2 OR SARS coronavirus 2 OR Severe Acute Respiratory Syndrome Coronavirus 2 OR severe acute respiratory syndrome) **AND** ((corona virus 2 OR new coronavirus) OR ((new AND Coronavirus) OR (novel coronavirus OR (novel AND corona virus) OR novel CoV OR (novel AND CoV) OR novel HCoV OR novel AND HCoV))))) OR ((“19″ OR “2019” OR Wuhan OR Hubei) **AND** (coronavirus* OR corona virus* OR CoV OR HCoV OR longCOVID* OR postCOVID* OR postcoronavirus* OR postSARS* OR coronavirus* OR corona virus*OR betacoronavirus*) **AND** (outbreak* OR epidemic* OR pandemic* OR crisis)) OR ((Wuhan OR Hubei) **AND** (pneumonia)) **AND** (2019/10/31:3000/12/31[Date – Publication])**)** **AND** (frail*)**)**

**CINAHL**

**((**((COVID-19 OR SARS-CoV-2 OR Coronavirus OR Betacoronavirus) **AND** (Disease Outbreak* OR Epidemic* OR Pandemic*)) OR ((nCoV OR 2019nCoV OR 19nCoV OR COVID19* OR COVID OR SARS-CoV-2 OR SARSCOV-2 OR SARS-COV2 OR SARSCOV2 OR SARS coronavirus 2 OR Severe Acute Respiratory Syndrome Coronavirus 2 OR severe acute respiratory syndrome) **AND** ((corona virus 2 OR new coronavirus) OR ((new AND Coronavirus) OR (novel coronavirus OR (novel AND corona virus) OR novel CoV OR (novel AND CoV) OR novel HCoV OR novel AND HCoV))))) OR ((“19″ OR “2019” OR Wuhan OR Hubei) **AND** (coronavirus* OR corona virus* OR CoV OR HCoV OR longCOVID* OR postCOVID* OR postcoronavirus* OR postSARS* OR coronavirus* OR corona virus*OR betacoronavirus*) **AND** (outbreak* OR epidemic* OR pandemic* OR crisis)) OR ((Wuhan OR Hubei) **AND** (pneumonia)) **AND** (2019/10/31:3000/12/31[Date – Publication])**)** **AND** (frail*)**)**
